# Teaching Presence, Self-Regulated Learning and Learning Satisfaction on Distance Learning for Students in a Nursing Education Program

**DOI:** 10.3390/ijerph19074160

**Published:** 2022-03-31

**Authors:** Leeho Yoo, Dukyoo Jung

**Affiliations:** College of Nursing, Ewha Womans University, Seoul 03760, Korea; haha_riho@naver.com

**Keywords:** education, distance, personal satisfaction, students, nursing

## Abstract

The novel coronavirus pandemic has dramatically affected how nursing students are educated. Distance learning has become the norm, and an evaluation of learning achievement is needed. This is a mixed-method study of teaching presence, self-regulated learning, and learning satisfaction in distance learning to evaluate the learning achievement of students in a nursing education program. Ninety-four students for quantitative and seven students for qualitative research were sampled. All the sampled students attend the nursing education program in Seoul and Gyeonggi Province and were enrolled during the first semester of 2020. Quantitative data were analyzed using SPSS/WIN 21.0, and qualitative data were analyzed via content analysis in NVivo 12. Teaching presence and self-regulated learning were identified as the factors affecting learning satisfaction. In a focus group interview, teaching presence increased when the students received feedback and saw the faces of their professors. Self-regulated learning occurred when they had opportunities to practice self-study and leadership and when they formed relationships between professors and colleagues. These methods have also been recognized to increase learning satisfaction. Considering the results of this study, it is necessary to develop teaching methods that enhance the learning satisfaction of students in distance learning nursing education programs.

## 1. Introduction

COVID-19, which started in December 2019 and eventually spread worldwide, caused people to distance themselves from others to avoid contamination. Hence, nursing education across South Korea conducts semesters through distance learning. Even before the COVID-19 pandemic, distance learning was already practiced abroad to overcome geographical limitations. However, in South Korea, online media were used in blended learning and flipped learning to assist face-to-face classes rather than distance learning [1,2]. However, as the COVID-19 pandemic continues, distance learning has already become increasingly common in the country. Studies also changed as COVID-19 continues. In the early days of COVID-19, qualitative studies related to the experience of distance learning were conducted [1], and now that distance learning has become more common, research on learning influencing factors is conducted [3,4].

With the unremitting COVID-19 pandemic, evaluation of learning outcomes, especially in regions where distance learning has become common, is increasingly needed to maintain the quality of education. Currently, the outcome of distance learning is measured as learning satisfaction [5] and learning achievement [6]. Learning satisfaction activates learning based on the subjective judgment of the learners [7]. It is a performance variable that evaluates the learning effect and the most basic performance indicator that affects learning achievement and learning persistence [8,9].

There are various factors affecting learning satisfaction in nursing distance learning: teaching presence [10], interaction [11], learner participation [12], self-regulated learning [13], and self-efficacy [14]. As various influencing factors were studied, a meta-analysis was also conducted on the influencing factors of learning satisfaction in distance learning.

In a meta-analysis about learning satisfaction on distance learning, the influencing factors were classified into external and internal factors, such as teaching environment and learning ability, which were presented as subfactors [7]. External and internal factors must be examined together to understand learning satisfaction as a whole. The teaching environment factor is about instructors and contents [7]. Teaching presence, which is a type of teaching environment, means the existence of learners’ feeling of interaction with instructors. This type plays a crucial role in distance learning where immediate interaction between instructors and learners is difficult [15]. Moreover, learning ability is a learning skill possessed by a learner in advance [7]. Self-regulated learning is a kind of learning ability described as self-controlled learning. It can be an important factor in distance learning considering that learners may choose their learning time, learning space, and learning progress [16].

Thus, teaching presence and self-regulated learning were studied as influencing factors of learning satisfaction in nursing distance learning. However, studies have not been conducted including both internal and external influencing factors of learning satisfaction. In addition, to present practical methods to increase learning satisfaction, researchers should conduct a quantitative study to understand how the internal and external influencing factors of learning satisfaction are related and how each factor affects learning satisfaction through a qualitative study. Therefore, a mixed-method study that understands research problems by interpretation based on both quantitative and qualitative research data is appropriate. Therefore, this study aimed to conduct mixed-method research on teaching presence, self-regulated learning, and learning satisfaction in nursing education to provide basic data that helps increase the learning satisfaction of distance learning.

## 2. Materials and Methods

### 2.1. Design

To identify in-depth characteristics and relationships between teaching presence, self-regulated learning, and learning satisfaction, we conducted a mixed-method study using focus group interview (FGI). The convergent design was used, collecting and analyzing each of the quantitative and qualitative data and integrating both data during discussion [17].

### 2.2. Participants

We enrolled nursing college undergraduate students who experienced distance learning in theoretical courses because of COVID-19 in Gyeonggi-do and Seoul in South Korea.

The sample size of the quantitative study was calculated using the G*Power 3.1 program. In line with a previous study [18], we assumed an effect size of 0.15, a significance level of 0.05, a statistical power of 0.80, and the number of predictors as 3 for multiple regression analysis. The minimum sample size required was 84 people. We recruited 94 participants, with consideration of a dropout rate of 10%. Ultimately, 94 participants were recruited. Seven participants who agreed to participate in FGI and recording were contacted. We included seven participants per group.

### 2.3. Data Collection

The quantitative study was conducted from 30 December 2020, to 15 February 2021, with convenient sampling. Before data collection, we contacted the professors at a nursing university in Gyeonggi-do and Seoul via e-mail to explain the purpose, content, and method of the study. After obtaining permission, we recruited participants, particularly those who accessed the URL of the online survey and agreed to participate in the study. The participants were asked to fill out the online questionnaire themselves. The questionnaire took approximately 10 min to complete.

The quantitative study was conducted from 30 December 2020, to 15 February 2021, with convenient sampling. Before data collection, we contacted the professors at a nursing university in Gyeonggi-do and Seoul via e-mail to explain the purpose, content, and method of the study. After obtaining permission, we recruited participants, particularly those who accessed the URL of the online survey and agreed to participate in the study. The participants were asked to fill out the online questionnaire themselves. The questionnaire took approximately 10 min to complete.

Considering the COVID-19 pandemic, the qualitative data were collected through FGI via ZOOM video conferencing. Before the meeting, the purpose and procedure of the study were explained to the participants through telephone, and then the consent URL was sent. A week before the interview, the interview questionnaire was sent to the participants via e-mail so that they could think in advance of what to discuss. Important contents were taken note of, and the researchers’ interpretation was verified by the participants, focusing on the written contents. The recorded interview was approximately 130 min long; on the same day, we transcribed the interview content to avoid missing the main information and vivid feeling at that time. The transcription was completed the next day.

### 2.4. Ethical Considerations

This study was conducted after obtaining approval from the Institutional Review Board (No. 202012-0003-02). Potential participants were asked to voluntarily click the online survey URL. In addition, the purpose, content, method, anonymity, confidentiality, and the possibility of withdrawal and suspension of the study were fully explained in the online consent form.

### 2.5. Measurement

#### 2.5.1. Demographic and Distance Learning Characteristics

The demographic and distance learning characteristics of nursing students included the age, sex, and semester in attendance, while the general characteristics of distance learning were the number of classes, the preference type of classes, and the characteristics of the distance learning platform.

#### 2.5.2. Teaching Presence

The teaching presence was measured using a tool developed by Akyol and Garrison [19] and was translated in Korean [20]. The tool consists of 12 questions, and the higher the score on a 5-point scale, the higher the sense of teaching reality. It is reliable, with a Cronbach’s α of 0.94; in this study, it was 0.86.

#### 2.5.3. Self-Regulated Learning

The self-regulated learning tool was measured using the online Self-Regulation Questionnaire (OSRQ) developed by Cho and Cho [21]. After the developers approved the use of the tool, one of us and a bilingual person fluent in Korean and English translated it into Korean by using a translation–reverse translation method. It consists of 11 questions focusing on learner–learning content interaction, 9 questions focusing on instructor–learner interaction, and 10 questions focusing on learner–learner interaction: the higher the score on a 7-point scale, the higher the degree of self-regulated learning. In terms of reliability, the Cronbach’s α was 0.94 at the time of development, and in this study, it was 0.91.

#### 2.5.4. Learning Satisfaction

The learning satisfaction tool is an 8-point scale developed by Kim and Park [22]; the higher the score, the higher the learning satisfaction. Regarding reliability, the Cronbach’s α was 0.96, and in this study, it was 0.94.

#### 2.5.5. Factors Influencing Learning Satisfaction

To confirm the meaning of teaching presence and self-regulated learning on learning satisfaction, we drafted a questionnaire and organized a final interview question, which was asked by one nursing professor. The main question of the FGI was “What do you think about the ‘feeling that the instructor is present’ in learning satisfaction during distance learning?,” and “What do you think of ‘doing your own learning’ in learning satisfaction during distance learning?”

### 2.6. Data Analysis

The collected quantitative data were analyzed using the SPSS/WIN 22.0 program (IBM Corp. Armonk, NY, USA). The significance level of was 0.05. The difference between teaching reality, self-regulated learning, and learning satisfaction in nursing students was evaluated by independent sample t-test, and their correlation was examined using Pearson’s correlation coefficient. The effect of teaching presence and self-regulated learning on learning satisfaction was confirmed by multiple regression analysis.

The recorded qualitative data were analyzed according to a traditional content analysis method, utilizing the NVivo 12 program. Traditional content analysis methods are used to describe phenomena, especially when existing theories or research literature are limited [23]. Given that distance learning research has been conducted since 2020 because of the COVID-19 pandemic, using the traditional content analysis seemed appropriate. In this analysis, we transcribed the interview contents while listening to the interview contents repeatedly, and then extracted meaningful statements from the recorded contents. Next, the extracted statements were repeatedly read, and common statements were categorized. Finally, the context was summarized by analyzing and integrating the classified categories.

## 3. Results

### 3.1. Quantitative Data

#### 3.1.1. Demographic Characteristics of Participants and the General Characteristics of Distance learning

We investigated 94 nursing students, with a mean age of 22.37 ± 3.24 years. On average, they took 4.55 classes, and 71.3% of them answered that real-time remote classes were the most preferred. The satisfaction rate of the distance learning platform was 3.51 ± 0.77 out of 5 points (Table 1). All FGI participants were women, two in the fourth semester, one in the sixth semester, and four in the eighth semester.

#### 3.1.2. Level of Teaching Presence, Self-Regulated Learning, and Learning Satisfaction

In terms of difference, the average sense of teaching presence was 3.66 ± 0.51 out of 5 points, whereas that of self-regulated learning was 5.15 ± 0.78 out of 7 points. Furthermore, self-regulated learning was 5.21 ± 0.69 out of 7 points compared with learning satisfaction, which was 3.76 ± 0.52 out of 5 points (Table 2).

#### 3.1.3. Correlation between Research Variables

The sense of teaching presence and self-regulated learning of nursing students (*r* = 0.51, *p* < 0.001) showed a statistically significant positive correlation with learning satisfaction (*r* = 0.72, *p* < 0.001). Likewise, a statistically significant positive correlation was found between self-regulated learning and learning satisfaction (*r* = 0.54, *p* < 0.001). Therefore, the higher the teaching presence and self-regulated learning were, the higher the learning satisfaction (Table 3).

#### 3.1.4. Effect of Teaching Presence and Self-Regulated Learning on Learning Satisfaction

In the multiple regression analysis, teaching presence (β = 0.60, *p* < 0.001, and variance inflator factor [VIF] = 1.35) and self-regulated learning (β = 0.23, *p* = 0.005, and VIF = 1.35) had a statistically significant effect on the learning satisfaction of nursing students in distance learning. Therefore, teaching presence and self-regulated learning may affect learning satisfaction. The explanatory power of the model was 54.3% (Table 4).

### 3.2. Qualitative Data

Through the traditional content analysis method, 4 themes, 10 categories, and 22 subcategories were formed from the FGI interview data of the nursing students. The derived topics were “convenience of distance learning”, “existence of instructors”, “Autonomy of students”, and “changes in distance learning” (Table 5).

#### 3.2.1. Convenience of distance learning

- Utilization of time and place

The designated place and class time of schools restricted students’ individual use of time. However, in distance learning, students had free time for activities other than study. Thus, the participants were satisfied with distance learning.

“Previously, I spent three hours to commute going to school. Now, I can use that time in other things.” (Participant 6)

“I think I was able to use my time more efficiently in the areas I’m interested in because I could control my learning time and personal time well through online classes.” (Participant 5)

- Efficient class

In distance learning, information search through electronic devices allows abundant use of class-related materials and sharing of opinions with fellow students through online chat increased understanding in the class. In addition, classes were conducted mostly without small talk, leading to an efficient class progress. Thus, learning satisfaction on remote classes increased.

“I was able to search and participate more diligently because I could search on the computer at the same time, and of course, it affects learning satisfaction, so I think I look back once more and focus more on learning content.” (Participant 1)

“In the case of recorded lectures, professors lead the contents without small chat, so I think that part was good.” (Participant 2)

#### 3.2.2. Existence of Instructors

- Feedback from instructors

Calling names, questioning, and facing the video make participants feel the presence of the instructors. The feedback was that feeling the presence of an instructor was helpful in their learning, resulting in class satisfaction.

“When I was in class, I think I felt like I was with the professor because he turned on the cam, other students turned on the cam, called me, and asked questions from time to time.” (Participant 5)

“I thought it was motivating in the classroom because I felt that I was not unilaterally provided with knowledge, but that it was achieved in two directions.” (Participant 3)

- Proper teaching methods

Holding a small meeting and timely help from instructors makes teaching presence, and it leads to learning satisfaction.

“Professor comes or keeps asking for opinions by holding a small meeting. I think the reality depends on how the professor does it.” (Participant 2) 

“I think that the right time and accurate announcements about class are helpful. Also, I think it will be helpful for students if they take classes and give appropriate assignments such as reflection papers.” (Participant 3)

#### 3.2.3. Autonomy of Students

- Self-planned learning

Participants achieved the independence of learning through planning distance learning and learning themselves. Additional studies can also be conducted as needed. Given that questions were recorded during class, they could review these questions one more time before answering them. Such self-learning increased their learning satisfaction.

“I think I liked the advantages of being able to replay in the case of parts that I couldn’t understand while taking the classes.” (Participant 6)

“In the case of recorded lectures, the time was set, so I thought it was a kind of initiative to plan well and use the time efficiently, and I thought it also led to the establishment of an environment where I could focus on the class.” (Participant 3)

“I had to write an email, cyber campus, and a text record, so I had to ask questions more carefully. I take the initiative in searching for books, browsing the Internet, and then asking questions…” (Participant 5)

- Negligent participation

Because of poor concentration and the questioning method, which was cumbersome, the participants could not take the lead in class.

“I have to ask the professor individually. But the mobile message looks rude, and the mail is a little cumbersome, I often use Internet instead of asking questions” (Participant 4)

“I have to participate only with my will without anyone. It was hard to do it with my own will.” (Participant 2)

- Leading the class

The participants experienced classes that reflected their opinions and classes with feedback, such as classes focusing on their own tasks. Consequently, leadership between instructors and learners can be achieved, thereby increasing learning satisfaction.

“I thought that I was leading the class when the professor considered me and collects students’ opinions.” (Participant 7) 

“When the class is centered on my assignment, I feel leading the class. I can share the screen; thus, I make an initiative in the class.” (Participant 1)

- Building relationships between students

Having the initiative to build peer relationships was more required in distance learning than in face-to-face classes. Distance learning restricted the participants from forming peer relationships. Despite not knowing each other, they had to have relational initiative, as needed, to complete their group assignments.

“We had to meet during our free time for group assignments, but since we filled those times with our own time, I think it was difficult to form a relationship.” (Participant 7)

“If I don’t contact colleagues first, there will be no meeting for group assignments. So, in distance learning, I thought initiative was an essential factor when teaming up.” (Participant 1)

#### 3.2.4. Changes in Distance Learning

- Advantage of face-to-face classes

The participants mentioned that in distance learning, tuition is a waste. They added that campus life is precious and that they want to return to face-to-face classes.

“I went to the central library to check a lot of major books or materials that I couldn’t purchase. But this semester, there are many cases where I couldn’t check the data. If I can’t use this, tuition is a waste…” (Participant 5)

“I want to enjoy a campus life that I can only enjoy at that time” (Participant 7)

- Necessity of supplementation

Considering that distance learning has limitations, participants wanted to reflect on the advantages of face-to-face classes and remote classes and apply them both.

“I hope it will be mixed so that people like me who are effective in taking classes in person can go and listen.” (Participant 1)

“In the case of practice, it would be nice to turn it into simulation 2–3 days out of 10 days to make use of the advantages of online that cannot be felt in the field.” (Participant 2)

## 4. Discussion

This research is a mixed-method study conducted on nursing students to understand the relationship and context between teaching presence, self-regulated learning, and learning satisfaction on distance learning. According to the results, the main influencing factors of learning satisfaction on distance learning were teaching presence and self-regulated learning, with an explanatory power of 54.3%, similar to the previous studies involving Korean cyber university students [24]. In web-based e-learning, teaching presence mediates between self-regulated learning and learning achievement [24]. Learning satisfaction highly correlates with learning achievement [25]. Considering the results of previous studies and this study, nursing students positively recognize teaching presence through self-regulated learning, leading to learning achievement and learning satisfaction. In our FGI, students’ opinions were reflected in distance learning; they could interact with instructors in classes centered on their tasks. In other words, students can experience teaching presence through self-regulated learning; thus, learning can be satisfactory for them.

Teaching presence in this study was higher than that in previous studies investigating animation-based web education [26]. To increase teaching presence, communication between instructors and students during lectures is essential [27], leading to different results from previous studies. Communication may reduce in animation-based web education. However, this study targeted real-time video lectures, pre-recorded lectures, and data presentation lectures. In addition, more than half of the study participants experienced real-time video lectures that could communicate, so the teaching presence was highly measured. Our FGI also revealed that showing the face of the instructor and having feedback were the main factors of teaching presence, consistent with previous studies which suggested that timely feedback, participation encouragement, content summary, and discussion reinforcement are the factors [26].

Self-regulated learning is divided into “learner-contents interaction”, “learner-instructor interaction”, and “learner-learner interaction” [21]. The FGI contents of this study also found that each aspect affects learning satisfaction differently. The results of the interview show “self-planned learning” in the “learner–content interaction” categories, “negligent participation” and “leading the class” in the “learner–instructor interaction” categories, and “building relationships between students” in the “learner–learner interaction” categories.

Self-regulated learning in the present study was lower than that in a U.S. study involving online pedagogy students [21]. Having the initiative to establish relationships with instructors is generally difficult. According to our FGI, when contacting the instructor, the mobile message seemed rude, and the mail was burdensome, indicating a sense of distance from the instructor. Accordingly, the need for a different platform to communicate with instructors was raised. Thus, platform satisfaction is less in the present study than in previous studies [28]. The more efficient the learning management system (LMS) is, the higher the instructor interaction and self-directed learning [29,30]. In addition, self-regulated learning elements were proposed to be included in the LMS, and instructors’ learning situation guidance function, achievement goal confirmation function, and immediate feedback function should be added [31]. Therefore, changing the LMS in the form of inducing student participation or increasing communication opportunities with instructors is necessary so that learners can have the initiative to form relationships with instructors.

According to our FGI, having a leading relationship between colleagues was essential to conduct classes, and it was intended to form a community between colleagues. To increase the initiative between learners in remote classes, there is the need to come up with an idea so that the number of contacts between learners increases. In previous studies, unlike face-to-face learning, distance learning required instructor intervention because interactions between learners did not occur naturally because of environmental constraints [32]. In addition, a structured cooperative learning strategy with a common goal is required to increase interaction between learners, and simultaneous interaction is effective [33]. Therefore, distance learning should focus on group tasks bututilize the function of small meetings, group meetings should be held within class hours to ensure simultaneous interaction.

In other words, learning satisfaction of nursing students on distance learning is influenced by teaching presence and self-regulated learning. Additionally, learning satisfaction can be achieved through timely feedback, use of the function of small meetings, and changes in class platforms.

This study has limitations. Given that it was conducted on nursing students enrolled in nursing colleges in Gyeongi-do and Seoul in South Korea, generalization of the research results is limited. In addition, the form of distance learning varies from real-time video lectures, pre-recorded lectures, and data-presenting lectures, and the difference between theoretical classes and nursing practice is also large; thus, caution should be taken when interpreting the results. Nevertheless, this study has identified the factors affecting the learning satisfaction of nursing students on distance learning, and the details were identified through FGI.

## 5. Conclusions

This study investigated the relationship between teaching presence, self-regulated learning, and learning satisfaction on distance learning among nursing students in Gyeonggi-do and Seoul in South Korea. A mixed-method study was conducted, integrating both quantitative and qualitative studies, and a convergent method was applied for a comprehensive analysis of research problems. Results showed that teaching presence and self-regulated learning influence learning satisfaction, and specific strategies were derived. Hence, this study can be used as a basis for preparing teaching methods suitable for distance learning for nursing students by promoting a comprehensive and in-depth understanding of teaching presence, self-regulated learning, learning satisfaction, and related factors. As a result of this study, providing appropriate feedback, assignments, and relationships can increase learning satisfaction. Therefore, it is necessary to study how to apply these teaching methods to practical and theoretical subjects of nursing students. In addition, an intervention study is needed to confirm whether this teaching method improves the learning satisfaction of nursing students. Policy and financial support should also be provided to properly establish a distance learning platform in universities to provide effective communications between users. Lastly, as teaching presence and self-regulated learning vary depending on the learner’s tendency, the varying tendency can affect learning satisfaction differently. Therefore, a follow-up study is needed to consider variables such as learner tendency.

## Figures and Tables

**Table 1:** Demographic characteristics of participants and the characteristics of distance learning. (*n* *=* 94).

Characteristics		*n*	%	M (±SD)
Years				22.37 (±3.24)
Sex	Male	5	5.3	
	Female	89	94.7	
Number of classes				4.55 (±1.16)
Preference type for distance learning	Real-time	67	71.3	
	Prerecorded	24	25.5	
	Data-presenting	3	3.2	
Satisfaction of LMS				3.51 (±0.77)

LMS: Learning Management System

**Table 2:** Level of teaching presence, self-regulated learning, and learning satisfaction. (*n* *=* 94).

Characteristics		M (±SD)	Min–Max
Teaching presence		3.66 (±0.51)	1–5
Self-regulated learning	Learner–contents	5.30 (±0.65)	1–7
	Learner–instructor	5.20 (±0.89)	
	Learner–learner	5.12 (±0.73)	
	Total	5.21 (±0.69)	
Learning satisfaction		3.76 (±0.52)	1–5

**Table 3:** Correlation between research variables. (*n* *=* 94).

	Teaching Presence	Self-Regulated Learning	Learning Satisfaction
	*r* *(p)*	*r* *(p)*	*r* *(p)*
Teaching presence			
Self-regulated learning	0.51 (<0.001)		
Learning satisfaction	0.72 (<0.001)	0.54 (<0.001)	

**Table 4:** Multiple regression analysis on learning satisfaction.

Characteristics	β	SE	*t*	*p*
Teaching presence	0.60	0.08	7.34	<0.001
Self-regulated learning	0.23	0.10	2.85	0.005
R^2^ = 0.55, adj. R^2^ = 0.54, F = 56.25, Sig.F < 0.001				

β: regression coefficient, SE:standard error of the regression coefficient.

**Table 5:** Learning satisfaction described by participants.

Theme	Categories	Subcategories
Convenience of distance learning	Utilization of time and place	Learning can be anywhere, any time
		Utilizing free time
	Efficient class	Utilizing electronic device
		Productive class
Existence of instructors	Feedback from instructors	Interaction and facing make teaching presence
		Feedback leads to learning satisfaction
	Proper teaching methods	Better interaction in small class
		Timely assistance from instructor
Autonomy of students	Self-planned learning	Repeated as needed
		Planning time and place for learning
		Additional self study as needed
	Negligent participation	Inconvenient Q&A systems
		Study alone
		Losing concentration
	Leading the class	Considering students’ opinions
		Classes which focus on making relationships
	Building relationships between students	Lack of opportunities to make relationships
		Make relationships as needed
Changes in distance learning	Advantage of face-to-face class	Waste of tuition
		Importance of campus life
	Necessity of supplementation	Change to blended learning
		Need to change teaching method

## Data Availability

The data are not publicly available because of privacy or ethical restrictions.
